# Severe Burn of the Neck, Treated by Extension from the Beginning

**Published:** 1860-04

**Authors:** 


*Severe Burn of the Neck, treated by extension from the beginning.*—On the 8th inst., a little girl, about nine years old was given chloroform at Guy's Hospital, and an effort was made by Mr. Hilton to flex her right arm, which had become anchylosed in consequence of a severe burn some two years ago. All his efforts were fruitless, because the union was now bony and extremely firm. Her case otherwise was a very remarkable and interesting one, from the fact of her complete recovery from a terrible burn of the whole anterior part of the neck and upper part of the chest, sustained two years ago, without the least deformity whatsoever.

When admitted into Guy's after the accident, the destruction of the skin was so extensive that the pectoral and some of the muscles of the neck were exposed, and the burn extended onwards to the right arm and forearm, whilst it occupied each side of the neck, involving the external surfaces of both of her ears. She was retained in bed for six months; her head and neck were kept perfectly quiet, and the latter was maintained in an extended position by means of a small bag of sand placed at the back of it. She was allowed, however, to sit up to her meals. When she left her bed she wore a stock, which greatly elevated the chin ; from its lower part were attached two cushions, which pressed over the upper part of the chest, and permitted a free motion, with an utter absence of deformity. The thin cicatrix glides with perfect ease over all the subjacent parts. This stock is to be continued for a sufficient time, to prevent all tendency to contraction. It is one of the most satisfactory cases of the kind in regard to the treatment, considering the extent and severity of the burn, which we ever remember to have witnessed.

